# Unidentified women in emergency services: care provided in public hospital emergency services

**DOI:** 10.1590/0034-7167-2025-0480

**Published:** 2026-08-21

**Authors:** Fernanda Coura Pena de Sousa, Fernanda Oliveira Santos, Giulia Mendes Curityba, Cínthia Neves Fonseca, Alanna Gomes da Silva, Allana dos Reis Corrêa

**Affiliations:** I Universidade Federal de Minas Gerais. Belo Horizonte, Minas Gerais, Brazil; II Fundação Hospitalar do Estado de Minas Gerais. Belo Horizonte, Minas Gerais, Brazil

**Keywords:** Women’s Health, Emergency Nursing, Patient Identification Systems, Violence Against Women, Emergencies., Mujeres, Servicio de Urgencia en Hospital, Atención Hospitalaria, Enfermería, Sistemas de Identificación de Pacientes.

## Abstract

**Objectives:**

to verify the associations between sociodemographic and clinical characteristics and the identification process of unidentified women in a public emergency service.

**Methods:**

a quantitative, descriptive, and retrospective study that analyzed 58 medical records of unidentified women treated between 2019 and 2022.

**Results:**

the mean age was 32.2 years, and 75.8% were of mixed race. Admissions occurred mainly in the early morning hours, and 48.2% were referred by the Mobile Emergency Care Service. Cases classified as orange/very urgent predominated, with assaults being the most prominent. Non-identification persisted in 70.6% of cases in the emergency room and in 62% until the outcome. Identification and clinical outcome showed a significant association (χ^2^=15.696; p=0.047), with a higher frequency of direct discharge and death among unidentified women.

**Conclusions:**

maintaining anonymity compromises patient safety, reinforcing the need to improve nursing care management with effective identification and screening strategies sensitive to social and clinical vulnerability.

## RESUMEN

**Objetivos:**

verificar las asociaciones entre las características sociodemográficas y clínicas y el proceso de identificación de mujeres no identificadas en un servicio público de emergencia.

**Métodos:**

estudio cuantitativo, descriptivo y retrospectivo que analizó 58 historias clínicas de mujeres no identificadas atendidas entre 2019 y 2022.

**Resultados:**

la edad promedio fue de 32,2 años y el 75,8% eran mestizos. Los ingresos se produjeron principalmente en la madrugada, y el 48,2% fueron derivados por el Servicio Móvil de Urgencias. Predominaron los casos clasificados como naranja/muy urgentes, siendo las agresiones las más frecuentes. La no identificación persistió en el 70,6% de los casos en urgencias y en el 62% hasta el desenlace. La identificación y el desenlace clínico mostraron una asociación significativa (χ^2^=15,696; p=0,047), con mayor frecuencia de altas directas y fallecimientos entre las mujeres no identificadas.

**Conclusiones:**

mantener el anonimato compromete la seguridad del paciente, lo que refuerza la necesidad de mejorar la gestión de la atención de enfermería con estrategias de identificación y detección eficaces que sean sensibles a la vulnerabilidad social y clínica.

## INTRODUCTION

The emergency room (ER) is a sector that operates continuously, dedicated to providing assistance to patients with or without life-threatening conditions, whose health problems require immediate intervention^(1)^. In this scenario, it is common in emergency services to encounter patients whose identity is unknown. Unidentified patients are those who arrive at the ER without any identification documents and whose identity has not been confirmed before the start of healthcare assistance^(2)^. As a result, international studies indicate that unidentified patients are more exposed to risks of medication errors, diagnostic delays and limitations in communication among teams, which directly affects care safety and continuity^(3,4)^, thus being considered a population at risk^(2-5)^.

Recognizing the risks associated with the absence of identifying data and its direct impact on care safety, the Brazilian National Patient Safety Program, established in 2013, sets as one of its fundamental goals the correct identification of patients, a central axis for healthcare safety^(4,5)^. The lack of confirmed identity directly compromises this goal and is recognized as a public health problem, as it negatively impacts both quality of care and the proper recording of clinical and administrative information^(4,5)^.

More than an administrative challenge, non-identification constitutes a marker of social and clinical vulnerability^(3)^. From a nursing care perspective, authors such as Ayres^(6)^ and Waldow^(7)^ highlight that vulnerability emerges from the interactions between social, political and subjective conditions, influencing care production and individual autonomy. In this scenario, women constitute a particularly vulnerable group among unidentified patients, due to structural inequalities and gender-based violence that permeate their life contexts^(2,8,9)^.

This vulnerability is also reflected in the available empirical evidence. In Brazil, a 2023 report indicated that women accounted for 9.6% of deaths among unidentified patients^(10)^. Similarly, an Indian study identified that 11.25% of unidentified patients admitted to a neurology service were female^(11)^. Although these findings highlight the significant presence of women among the unidentified, national scientific production on the subject is incipient, and international literature still addresses it in a predominantly normative way, without delving into the relationships between lack of identity, gender, social vulnerability, and institutional invisibility in the context of emergency care.

The scarcity of studies with a critical approach to women in this condition reinforces an institutional invisibility that transcends the absence of a name or document. Without nominal recognition, their clinical trajectories become fragmented, and the gender specificities that permeate their illness remain hidden. Understanding socioeconomic conditions, the reasons for seeking care, the nature of the care provided, and the outcomes is fundamental to revealing the structural flaws that perpetuate inequalities in care. Therefore, this study formulated the following guiding question: what are the characteristics and associations of these with the identification process of unidentified women treated in a large public ER?

Analyzing the characteristics of care provided and its relationship to the process of identifying unidentified women in ERs allows us to understand not only their health needs, but also the structural failures that compromise care safety and care comprehensiveness. The lack of identification exposes these women to additional risks, such as difficulty in recognizing allergies and the impossibility of contacting family members, in addition to hindering the tracking of situations of violence and social vulnerability^(3,7-10)^. By illuminating these dimensions, this study contributes to the debate on patient safety from a gender perspective, reinforcing the ethical responsibility of nursing and multidisciplinary teams in building safer, more equitable, and humanized practices.

## OBJECTIVES

To investigate the associations between sociodemographic and clinical characteristics and the identification process of unidentified women in a public hospital emergency service.

## METHODS

### Ethical aspects

The ethical precepts described in Resolution 466/2012 of the Brazilian National Health Council were respected. This study is part of a larger project that analyzed unidentified patients regardless of gender. The research was approved by the *Universidade Federal de Minas Gerais* Research Ethics Committee, obtaining a favorable opinion. Due to the nature of the research, it was not possible to establish telephone contact with patients to apply the Informed Consent Form (ICF). Thus, the study obtained a waiver of the consent declaration. The researchers formalized the commitment to record-keeping with the co-participating institution through a Data Use Commitment Agreement and the application of the Justification for Waiver of ICF.

### Study design, period, and location

This is a quantitative, descriptive, and retrospective study, conducted between January 1, 2019, and December 31, 2022. The choice of this time frame was based on the occurrence of the COVID-19 pandemic, allowing for a comprehensive analysis of the different phases of the pandemic (before, during, and after). The study was developed in an ER of a large public hospital in Belo Horizonte, Minas Gerais, part of the Brazilian Health System. This hospital is classified as a Trauma Center I, representing an important referral center for polytrauma patients, urgent cases, and clinical emergencies in the state of Minas Gerais^(12)^.

The study design was guided by STrengthening the Reporting of OBservational studies in Epidemiology recommendations.

### Population and sample

The sample for this study comprised 58 women, all over 18 years of age, who sought care at the ER of the institution in question and who did not present identification documents upon admission. Patients who initially sought care at another health unit were excluded. This exclusion is due to the fact that, in these cases, initial care was not provided at the ER of the institution in question, making it impossible to perform a standardized analysis of admission conditions, risk stratification, and initial procedures, as well as introducing potential bias related to prior interventions that are not controllable within the scope of this study.

Between January 1, 2019, and December 31, 2022, the preliminary survey identified 2,425 unidentified patient visits to an ER. From this total population, a sample size calculation was performed considering a proportion for a finite population, a 5% margin of error, and a 95% confidence level, resulting in a sample of 332 randomly selected medical records. This calculation was originally designed for the overall analysis of unidentified patients, regardless of sex.

Among the 332 randomly selected medical records, 271 corresponded to male patients and 61 to female patients. Considering that this article focuses specifically on the analysis of unidentified women, only female records were eligible for this analysis. After applying the inclusion and exclusion criteria, three women were excluded because they were under 18 years of age, resulting in a final sample of 58 unidentified women.

### Study variables

A structured data collection form was used, which gathered the following variables, based on literature regarding patient care in the ER setting^(13-14)^: sociodemographic data (sex, age, race/color, type of residence); admission characteristics (time, day, month, referral origin, clinical priority according to the Manchester Triage System (MCS)); care characteristics (triage flowcharts and discriminators, care area, first specialty involved, admission diagnosis); initial clinical assessment (ABCDE approach, Glasgow Coma Scale score, length of stay in the ER, discharge status, time and method of identification). To express patients who were discharged directly from the ER, without being seen or referred to another hospital sector, we created the variable “direct discharge”, which includes hospital discharge and discharge at patients’ request.

To ensure data collection instrument validity, a pilot test was conducted with five medical records from different years of the study, selected randomly.

### Study protocol

Data collection was carried out in two phases. Initially, the number of records of unidentified patients was collected using data provided by the Medical Records and Statistics Service of the hospital in question and access to the institutional folder “Unidentified Patients”, upon authorization from those responsible. Subsequently, the medical records that comprised the sample were randomly selected using Minitab 17, selecting all records and randomly assigning the number of sample units for each year analyzed.

In the second phase, data were extracted from electronic medical records using the hospital’s computerized system. The dataset (sociodemographic data, initial assessment, care procedures upon admission, outcomes, and patient identification flow) derived from the electronic medical record was imported directly into Research Electronic Data Capture.

### Analysis of results and statistics

The database was exported and subjected to a verification process using Microsoft Excel^®^, ensuring the identification and correction of any inconsistencies to guarantee data accuracy before analysis.

Due to the exploratory nature of the study and the sample size (n=58), descriptive and bivariate analyses were chosen. Continuous variables were described using measures of central tendency and dispersion, employing the median, interquartile range (IQR), minimum and maximum values, due to the asymmetry observed in the distributions. Data normality was assessed using the Shapiro-Wilk test. For categorical variables, absolute and relative frequencies were calculated.

The comparison between the “identified” and “unidentified” groups was conducted using Pearson’s chi-square test, with Fisher’s exact test applied in situations of low expected cell counts. For comparison of continuous variables between two independent groups, the Mann-Whitney test was used, considering the absence of a normal distribution. The significance level adopted in all analyses was 5% (p<0.05).

## RESULTS

### Sociodemographic characteristics

Among the 58 unidentified women admitted to the ER, the age of 41.3% was recorded, ranging from 19 to 63 years, with a mean of 32.21 (standard deviation ±12.50). Concerning race, reported by third parties, the majority (75.8%) were classified as mixed-race. Place of residence was recorded in 43.1% of records, with more than half (60%) residing in Belo Horizonte. Of the 26 (44.8%) data obtained on type of housing, house and apartment were the majority (84.6%) (Table 1).

**Table 1:** Sociodemographic characterization of unidentified women admitted to the emergency room, Belo Horizonte, Minas Gerais, Brazil, 2019-2022

Variable	n	%
**Age (years)**	**24**	**100**
18 to 29	13	54.17
30 to 39	4	16.67
40 to 49	4	16.67
50 to 59	2	8.33
60 or more	1	4.17
**Race/color**	**58**	**100**
Brown	44	75.86
White	7	12.07
Black	7	12.07
**City**	**25**	**100**
Belo Horizonte	15	60
Metropolitan Region	8	32
Minas Gerais, outside MRBH	2	8
**Type of housing**	**26**	**100**
House/apartment	22	84.62
Homeless situation	4	15.38

*MRBH - Metropolitan Region of Belo Horizonte.*

### Characteristics of admission to the emergency room

October stood out for the highest frequency of admissions (17.2%), with Sundays (29.3%) being the days of the week with the most admissions, followed by Tuesdays (20.6%) and Saturdays (17.2%). In relation to hiring shift, the early morning period stood out among the others, with 34.4%, and the night period represented 31%, followed by the afternoon (24.1%) and morning (10.3%) periods.

As for the mode of arrival at the ER, transport was mainly by teams from the Mobile Emergency Care Service (48.2%), followed by private means (22.4%), public security agencies (12%), private ambulance (12%), and the general public (5.1%). Concerning the MTS data, 57 (98.3%) of patients were classified, with the most frequent priority level being orange/very urgent (63.2%), followed by red/emergency (21%). The most accessed flowchart was major trauma (40.3%), followed by assault (21%) (Table 2). The most frequent discriminators were significant trauma mechanism (36.8%), the combination of altered level of consciousness + altered level of consciousness not entirely attributable to alcohol + altered level of consciousness entirely attributable to alcohol (22.8%), and inadequate breathing (17.5%) (Table 2).

**Table 2:** Risk stratification data according to the Manchester Triage System for unidentified women admitted to the emergency room, Belo Horizonte, Minas Gerais, Brazil, 2019-2022

Variable	n	%
**Priority level - MTS result**	**57**	**100**
Red/emergency	12	21
Orange/very urgent	36	63.2
Yellow/urgent	6	10.5
Green/not very urgent	3	5.3
**MTS flowchart**	**57**	**100**
Major trauma	23	40.3
Assault	12	21
Falls	4	7
Adult malaise	4	7
Burns	2	3.5
Traumatic brain injury	2	3.5
Seizures	2	3.5
Apparent intoxication	2	3.5
Problems with extremities	2	3.5
Behavioral changes	1	1.8
Self-harm	1	1.8
Wounds	1	1.8
Overdose and poisoning	1	1.8
**MTS discriminator**	**57**	**100**
Significant trauma mechanism	21	36.8
Altered level of consciousness + altered level of consciousness not entirely attributable to alcohol + altered level of consciousness entirely attributable to alcohol	13	22.8
Inadequate breathing	10	17.5
Severe pain + moderate pain + recent mild pain	6	10.5
Sudden onset	2	3.5
Gross deformity	1	1.8
History of overdose or poisoning	1	1.8
History of trauma	1	1.8
New abnormal pulse	1	1.8
Airway obstruction	1	1.8
**Reason that brought patients to the emergency room**	**58**	**100**
Assault	17	29.3
Traffic accident	12	20.7
Fall	9	15.5
Run over	8	13.8
Exogenous poisoning	5	8.7
Altered level of consciousness	3	5.1
Burn	2	3.4
Seizure	2	3.4

SMCR - Sistema Manchester de Classificação de Risco;

* Agressão inclui agressão física, ferimento por arma de fogo, ferimento por arma branca;

** Acidente de trânsito inclui acidente automobilístico, acidente de motocicleta, acidente de bicicleta.

The most relevant reason that led women to the ER was assault (29.4%), including physical assault (12.1%), gunshot wound (5.2%), and stab wound (12.1%). Subsequently are traffic accidents (20.7%), comprising car accidents (12.1%), motorcycle accidents (6.9%), and bicycle accidents (1.7%) (Table 2).

### Outcome of care provided to unidentified women in the emergency room

Of the 58 cases analyzed, it was possible to record the outcome for 57 (98.3%) women. More than half of them (54.4%) did not require hospitalization, with a length of stay in the ER between zero and three days (median of zero; IQR of one day). Direct discharge from the service was the most frequent outcome, occurring in 48.2% of cases, and 6.9% of patients required outpatient follow-up. Among the other outcomes, 18.9% were referred to the surgical unit; 10.3% were hospitalized; 6.9% died; 6.9% requested discharge at their own request; 5.1% were transferred; and 1.7% left the service.

### Patient identification process

The length of time women remained “unidentified” ranged from zero to 16 days, with a median of zero days and an interquartile range of one.

Regarding the identification process, 22 (37.9%) patients were identified by the length of hospital discharge. Of these, 17 (77.3%) were identified while still in the ER. Data on the identification process are presented in Table 3.

**Table 3:** Process for identifying unidentified women admitted to the emergency room, Belo Horizonte, Minas Gerais, Brazil, 2019-2022

Variável	n	%
**Identification at some point during the stay in the ER**	**58**	**100**
No	41	70.7
Yes	17	29.3
**Identification up to the point of hospital outcome**	**58**	**100**
Yes	22	37.9
No	36	62.1
**Responsible for patient identification**	**22**	**100**
Family members	16	72.7
Self-identification	3	13.6
Friends	2	9.1
Police service	1	4.6
**Method used to identify patients**	**22**	**100**
Presentation of identification document	19	86.4
Self-declaration (pandemic)	3	13.6
**Sector in which patient was identified**	**22**	**100**
Emergency room	17	72.7
Surgical block	2	13.6
Inpatient unit	2	9.1
ICU	1	4.5
**Reasons for non-identification**	**36**	**100**
No reason recorded	15	41.7
Self-declaration without supporting documentation	13	36.1
Death	5	13.9
Refusal to identify	2	5.6
Refusal to contact family	1	2.7

*ER - Emergency Room; ICU - Intensive Care Unit.*

When comparing the care provided to identified and unidentified women during their stay in the ER, a significant association was observed between identification and clinical outcome. The distributions of outcomes differed significantly among groups (χ^2^=15.696; p=0.047), as shown in Table 4.

**Table 4:** Association between patient identification and emergency room outcome, Belo Horizonte, Minas Gerais, Brazil, 2019-2022

Outcome in the emergency room	Unidentified n (%)	Identified n (%)	Total n (%)
Direct discharge	26 (63.5)	6 (35.3)	24 (41.4)
Surgical block	8 (19.5)	3 (17.6)	11 (19.0)
Inpatient unit	1 (2.4)	5 (29.4)	6 (10.3)
Hospital transfer	1 (2.4)	2 (11.8)	3 (5.2)
Death	3 (7.3)	1 (5.9)	4 (6.9)
Escape	1 (2.4)	1 (5.9)	2 (3.4)
Medical record without resolution	1 (2.4)	0 (0.0)	1 (1.7)
**Total**	**41 (100)**	**17 (100)**	**58 (100)**

## DISCUSSION

It was identified that the most affected age group was predominantly young adult women, and the majority were registered by third parties as being of mixed race. This age range is explained by the fact that women who are victims of violence are generally in their reproductive and productive age range^(15)^. Furthermore, the Pan American Health Organization states that inequality is the main risk factor for violence against women, which explains how younger women, especially in lowand middle-income countries, face greater economic and social difficulties^(16)^. Factors such as low levels of education, lack of access to financial resources, and dependence on external partners contribute to greater vulnerability to abuse^(9,10)^.

Regarding race, a cross-sectional study using data from the VIVA Survey in 2011, 2014, and 2017 showed that the results reflect the Brazilian female population’s situation, since, in all three editions analyzed, the mixed-race population was the most affected in cases of assaults requiring urgent and emergency care^(17)^. In general, race/color is an important factor in women’s lives, reinforcing a context of racial inequality and social vulnerability that negatively impacts their health and access to medical services^(10)^.

The high proportion of missing data in the age and place of residence records of unidentified patients also deserves highlighting. This finding reflects the fragility of the registration and communication processes among teams, interfering with continuity of care and epidemiological surveillance. The absence of basic information prevents the recognition of territorial patterns of vulnerability, hindering the planning of public policies for prevention and social protection. Furthermore, it compromises nursing practices based on comprehensive care and patient safety, as it limits the contextual knowledge necessary for assertive clinical and social decisions.

Most patients were classified as urgent (orange priority), indicating clinical severity upon arrival at the service. This profile is consistent with studies that point to unidentified patients as a group at higher risk and severity in ERs^(2)^. The main flowcharts and discriminators in the reception process were related to trauma and altered levels of consciousness, frequently associated with episodes of aggression and alcohol use^(18)^. Assault was the most frequent reason for seeking medical attention, followed by traffic accidents and falls. In the case of assaults, the literature indicates a predominance of cases involving intimate partners, frequently with the use of physical force and, to a lesser extent, firearms or sharp objects^(9,19)^.

Given this scenario of high severity and prevalence of external causes, the role of nursing becomes essential from the first contact with the patient. The results highlight the relevance of nursing in welcoming and classifying the risk of unidentified women, especially given the high proportion of cases classified as orange and red priority. In the MTS, the absence of identifying information imposes additional challenges, requiring qualified listening and careful observation to avoid underestimating the risk^(18-20)^. In this context, nurses act as a link between clinical care and social protection, ensuring safe care, timely referrals, and coordination with the support network for women in vulnerable situations^(15,21)^.

From admission to outcome in the ER, only a portion of women were properly identified, while the majority remained anonymous throughout their care. Analysis of clinical outcomes revealed that the lack of identification negatively impacts care flow, as unidentified women had a higher proportion of direct discharges and deaths, as well as a lower probability of hospitalization. This pattern does not indicate effective resolution, but highlights limitations in the care provided to patients whose identity is not confirmed, whether due to clinical severity preventing communication or institutional failures in registration, reception, and intersectoral coordination processes. Anonymity, therefore, increases institutional invisibility and compromises continuity of care, hindering activation of support networks and more comprehensive clinical decision-making.

From the perspective of the ethics of care, the absence of recognized identity implies not only an administrative risk, but also a disruption in continuity of care and social protection. Nursing literature highlights that ethical care requires recognition and accountability for the other, overcoming institutional barriers that reinforce inequalities and invisibility^(7-9)^. Patients who leave the service without any form of identification remain outside the reach of surveillance, protection, and follow-up networks, which hinders the activation of public policies to combat violence and provide psychosocial support^(19,21)^.

In this context, nursing plays a central role as it is on the front line of welcoming, qualified listening, and intersectoral coordination. Nurses can intervene through expanded screening with a social focus, systematic recording of signs of vulnerability, and notification of suspected cases of violence, as recommended by the Technical Standard for Prevention and Treatment of Harm Resulting from Sexual Violence against Women and Adolescents^(21)^ and reinforced by studies on the role of nursing in addressing violence and patient safety^(21-23)^_._

Coordination with social services and women’s protection agencies, even after clinical stabilization, is fundamental to ensuring post-discharge linkage and integration into the psychosocial care network and the fight against violence. Thus, the association between time without identification and a lower chance of hospitalization not only highlights inequalities in the care flow but also calls on nursing to rethink its practices from an ethical and social justice perspective, guided by the principles of comprehensiveness, equity, and safety of care^(5,7,8)^.

### Study limitations

This study has some limitations that should be considered. Since it was conducted in a single large, specialized hospital, the results cannot be generalized to other healthcare settings. The retrospective nature of the research, based on electronic medical records, also imposed restrictions, as some of the clinical and social information was recorded in free text format, which made standardization and in-depth analysis of the data difficult. Furthermore, freehand writing is subject to interpretative variations, influenced by the subjectivity of the professionals who make the records, which can introduce bias into the findings.

Furthermore, incomplete or missing fields were observed in essential variables, such as age and place of residence, reflecting weaknesses in the registration processes and potentially limiting the completeness of the analysis. This characteristic is inherent to documentary studies and can introduce information bias. On the other hand, these gaps highlight a relevant aspect of the study’s own object: the precariousness of records on unidentified women, which in itself reinforces the discussion about vulnerability and invisibility in the hospital context.

### Contributions to nursing, health, or public policy

This study makes a significant contribution by highlighting a topic that has been little addressed in the field of urgent and emergency care: the care of unidentified women. By describing the clinical, social, and care flow characteristics of this population, the research broadens the understanding of their vulnerabilities and care gaps, especially in the processes of reception and risk stratification.

The findings reinforce the central role of nurses in triage, clinical judgment, and decision-making in emergency settings, where the lack of identification demands ethical sensitivity and technical discernment. The predominance of orange and red priorities, associated with cases of assault and trauma, indicates that nurses are frequently the first to recognize signs of violence and vulnerability, activating safety protocols, recording information, and referring the case to the protection network. Thus, the study provides support for improving care protocols and strengthening public policies focused on patient safety and comprehensive care for women, reaffirming nursing’s commitment to equitable, ethical care centered on human needs, even in the absence of basic information such as name or identification document.

## CONCLUSIONS

Unidentified women treated in public ERs were predominantly young adults of mixed race, with more frequent visits during the early morning hours and on weekends. Cases of assault and accidents were the main causes of admission, and most patients were classified as priority 2 (orange/very urgent) or 1 (red/emergency), indicating a high clinical risk and need for immediate intervention. It was observed that the lack of identification negatively impacts the care flow, as unidentified women had a higher proportion of direct discharges and deaths, and a lower probability of hospitalization.

The findings show that the lack of identification goes beyond an administrative issue, constituting a marker of social and care vulnerability that compromises care safety and continuity. In this context, nurses play a central role in welcoming and risk stratification, combining clinical judgment and sensitivity to recognize signs of violence, neglect, and social vulnerability. These results reinforce the need for institutional protocols and workflows that incorporate clinical and social criteria into the screening process, as well as safe, agile, and interdisciplinary identification strategies. Together, these elements strengthen ethical and humanized nursing practice, highlighting that the care of unidentified women requires an integrated and equity-oriented approach.

## Data Availability

The research data are available in a repository: https://doi.org/10.48331/SCIELODATA.NH50AO.
