# Supplementary material for: Unidentified women in emergency services: care provided in public hospital emergency services
Source: Rev Bras Enferm. 2026 Aug 21;79:e20250480. doi: 10.1590/0034-7167-2025-0480 (PMC13505556; doi:10.1590/0034-7167-2025-0480)
Supplement: Translation

# Translation: Mulheres não identificadas na assistência hospitalar: atendimentos em serviço hospitalar de emergência público

**DOI:** 10.1590/0034-7167-2025-0480pt

**Authors:** Fernanda Coura Pena de Sousa, Fernanda Oliveira Santos, Giulia Mendes Curityba, Cínthia Neves Fonseca, Alanna Gomes da Silva, Allana dos Reis Corrêa

**Affiliations:** I Universidade Federal de Minas Gerais. Belo Horizonte, Minas Gerais, Brasil; II Fundação Hospitalar do Estado de Minas Gerais. Belo Horizonte, Minas Gerais, Brasil

**Keywords:** Mulheres, Serviço Hospitalar de Emergência, Assistência Hospitalar, Enfermagem, Sistema de Identificação de Pacientes.

## Abstract

**Objetivos:**

verificar as associações entre características sociodemográficas, clínicas e o processo de identificação de mulheres não identificadas em um serviço público de emergência.

**Métodos:**

estudo quantitativo, descritivo e retrospectivo, que analisou 58 prontuários de mulheres não identificadas atendidas entre 2019 e 2022.

**Resultados:**

a média de idade foi de 32,2 anos, e 75,8% eram pardas. As admissões ocorreram sobretudo na madrugada, e 48,2% foram encaminhadas pelo Serviço de Atendimento Móvel de Urgência. Predominaram casos classificados como laranja/muito urgente, com destaque para agressões. A não identificação persistiu em 70,6% no pronto-socorro e em 62% até o desfecho. Identificação e desfecho clínico apresentaram associação significativa (χ^2^=15,696; p=0,047), com maior frequência de altas diretas e óbitos entre não identificadas.

**Conclusões:**

a permanência do anonimato compromete a segurança do paciente, reforçando a necessidade de aprimorar a gestão do cuidado de enfermagem, com estratégias eficazes de identificação e triagem sensível à vulnerabilidade social e clínica.

## INTRODUÇÃO

O pronto-socorro (PS) é um setor que funciona ininterruptamente, dedicado a prestar assistência aos pacientes com ou sem risco de morte, cujos agravos à saúde exigem intervenção imediata^(1)^. Nesse cenário, é comum nos serviços de emergência encontrar pacientes cuja identificação é desconhecida. Pacientes não identificados são aqueles que chegam sem nenhum documento de identificação no PS e que não tiveram sua identidade confirmada antes do início da assistência à saúde^(2)^. Em virtude disso, estudos internacionais apontam que pacientes não identificados estão mais expostos a riscos de erros de medicação, atrasos diagnósticos e limitações na comunicação entre equipes, o que afeta diretamente a segurança e continuidade da assistência^(3,4)^, sendo, assim, considerada uma população de risco^(2-5)^.

Reconhecendo os riscos associados à ausência de dados identificadores e seu impacto direto na segurança do cuidado, o Programa Nacional de Segurança do Paciente, instituído no Brasil em 2013, estabelece como uma de suas metas fundamentais a correta identificação do paciente, eixo central para a segurança assistencial^(4,5)^. A falta de identidade confirmada compromete diretamente essa meta, e é reconhecida como um problema de saúde pública, pois impacta negativamente tanto a qualidade do cuidado quanto o registro adequado das informações clínicas e administrativas^(4,5)^.

Mais do que um desafio administrativo, a não identificação constitui um marcador de vulnerabilidade social e clínica^(3)^. Na perspectiva do cuidado em enfermagem, autores como Ayres^(6)^ e Waldow^(7)^ destacam que a vulnerabilidade emerge das interações entre condições sociais, políticas e subjetivas, influenciando a produção do cuidado e a autonomia dos sujeitos. Nesse cenário, as mulheres compõem um grupo especialmente vulnerável entre os pacientes não identificados, em razão das desigualdades estruturais e da violência de gênero que perpassam seus contextos de vida^(2,8,9)^.

Essa condição de vulnerabilidade também se reflete nas evidências empíricas disponíveis. No Brasil, boletim de 2023 indicou que as mulheres corresponderam a 9,6% dos óbitos entre pacientes sem identificação^(10)^. De modo semelhante, estudo indiano identificou que 11,25% dos pacientes não identificados admitidos em um serviço de neurologia eram do sexo feminino^(11)^. Apesar de esses achados evidenciarem a presença significativa de mulheres entre os não identificados, a produção científica nacional sobre o tema é incipiente, e a literatura internacional ainda o aborda de forma predominantemente normativa, sem aprofundar as relações entre ausência de identidade, gênero, vulnerabilidade social e invisibilidade institucional no contexto do cuidado em emergência.

A escassez de estudos com abordagem crítica sobre as mulheres nessa condição reforça uma invisibilidade institucional que transcende a ausência de nome ou documento. Sem reconhecimento nominal, suas trajetórias clínicas se tornam fragmentadas, e as especificidades de gênero que permeiam seu adoecimento permanecem ocultas. Compreender as condições socioeconômicas, as causas de atendimento, a natureza da assistência prestada e os desfechos é fundamental para revelar as falhas estruturais que perpetuam desigualdades no cuidado. Diante disso, este estudo formulou a seguinte questão norteadora: quais as características e as associações destas com o processo de identificação de mulheres não identificadas atendidas em um PS público de grande porte?

Analisar as características dos atendimentos e suas relações com o processo de identificação de mulheres não identificadas em PS permite compreender não apenas suas necessidades de saúde, mas também as falhas estruturais que comprometem a segurança assistencial e a integralidade do cuidado. A ausência de identificação expõe essas mulheres a riscos adicionais, como dificuldade de reconhecimento de alergias e impossibilidade de contato com familiares, além de dificultar o rastreamento de situações de violência e vulnerabilidade social^(3,7-10)^. Ao iluminar essas dimensões, este estudo contribui para o debate sobre a segurança do paciente sob a ótica de gênero, reforçando a responsabilidade ética da enfermagem e das equipes multiprofissionais na construção de práticas mais seguras, equitativas e humanizadas.

## OBJETIVOS

Verificar as associações entre características sociodemográficas, clínicas e o processo de identificação de mulheres não identificadas em um serviço hospitalar público de emergência.

## MÉTODOS

### Aspectos éticos

Os preceitos éticos descritos na Resolução n^o^ 466/2012 do Conselho Nacional de Saúde foram respeitados. Este estudo faz parte de projeto maior que analisou os pacientes não identificados independente do gênero. A pesquisa foi aprovada pelo Comitê de Ética em Pesquisa da Universidade Federal de Minas Gerais, obtendo parecer favorável. Devido à natureza da pesquisa, não foi possível estabelecer contato telefônico com os pacientes para aplicação do Termo de Consentimento Livre e Esclarecido (TCLE). Assim, o estudo obteve dispensa da declaração de consentimento. Os pesquisadores formalizaram o compromisso dos registros com a instituição coparticipante mediante Termo de Compromisso de Utilização de Dados e a aplicação do Termo de Justificativa de Dispensa do TCLE.

### Desenho, período e local do estudo

Trata-se de estudo quantitativo, descritivo e retrospectivo, realizado no período de 1 de janeiro de 2019 a 31 de dezembro de 2022. A escolha deste intervalo temporal se fundamentou na ocorrência da pandemia de COVID-19, possibilitando a análise abrangente das distintas fases da referida pandemia (prévia, durante e subsequente). O estudo foi desenvolvido em um PS de um hospital público de grande porte, em Belo Horizonte, Minas Gerais, inserido no Sistema Único de Saúde. Esse hospital é classificado como Centro de Trauma I, representando importante referência no atendimento a pacientes politraumatizados, urgências e emergências clínicas no estado de Minas Gerais^(12)^.

As recomendações do *STrengthening the Reporting of OBservational studies in Epidemiology* nortearam o desenho do estudo.

### População e amostra

Compuseram a amostra deste estudo 58 mulheres, todas maiores de 18 anos, que procuraram atendimento no PS da instituição cenário e que não apresentavam documento de identificação à admissão na unidade. Foram excluídas as pacientes que inicialmente buscaram atendimento em outra unidade de saúde. Essa exclusão se deve ao fato de que, nesses casos, o atendimento inicial não foi realizado no PS da instituição cenário, impossibilitando a análise padronizada das condições de admissão, da classificação de risco e das condutas iniciais, além de introduzir potencial viés relacionado a intervenções prévias não controláveis no escopo deste estudo.

No período de 1 de janeiro de 2019 a 31 de dezembro de 2022, o levantamento preliminar identificou 2.425 atendimentos a pacientes não identificados no PS. A partir dessa população total, foi realizado o cálculo amostral considerando proporção para população finita, margem de erro de 5% e nível de confiança de 95%, resultando em uma amostra de 332 prontuários, sorteados aleatoriamente. Esse cálculo foi originalmente concebido para a análise global de pacientes não identificados, independentemente do sexo.

Entre os 332 prontuários sorteados, 271 correspondiam a pacientes do sexo masculino, e 61, a pacientes do sexo feminino. Considerando que o presente artigo tem como foco específico a análise das mulheres não identificadas, apenas os prontuários do sexo feminino foram elegíveis para esta análise. Após a aplicação dos critérios de inclusão e exclusão, três mulheres foram excluídas por apresentarem idade inferior a 18 anos, resultando em uma amostra final de 58 mulheres não identificadas.

### Variáveis do estudo

Foi utilizado um formulário estruturado de coleta de dados que reuniu as seguintes variáveis, fundamentadas nas literaturas referentes ao atendimento a pacientes no ambiente de PS^(13,14)^: dados sociodemográficos (sexo, idade, raça/cor, tipo de residência); características da admissão (horário, dia, mês, origem do encaminhamento, prioridade clínica conforme o Sistema Manchester de Classificação de Risco (SMCR); características do atendimento (fluxogramas e discriminadores de triagem, área de atendimento, primeira especialidade envolvida, diagnóstico de admissão); avaliação clínica inicial (abordagem ABCDE, escore da Escala de Coma de Glasgow, tempo de permanência no Departamento de Emergência, *status* de alta, momento e método de identificação). Para expressar as pacientes que receberam alta direta do PS, sem serem atendidas ou encaminhadas para outro setor do hospital, criamos a variável “alta direta”, que inclui a alta hospitalar e a alta a pedido.

Para garantir a validade do instrumento de coleta de dados, foi realizado teste piloto com cinco prontuários de diferentes anos do estudo, selecionados aleatoriamente.

### Protocolo do estudo

A coleta de dados foi realizada em duas fases. Inicialmente, foi efetuado o levantamento dos números de registros de pacientes não identificados por meio de dados fornecidos pelo Serviço de Arquivo Médico e Estatística do hospital cenário e do acesso à pasta institucional “Pacientes Não Identificados”, após autorização dos responsáveis. Em seguida, procedeu-se ao sorteio dos prontuários, que compuseram a amostra a partir do *software* Minitab 17, de forma que selecionasse todos os prontuários e realizasse o sorteio da quantidade de unidades amostrais para cada ano analisado.

Na segunda fase, foram extraídos dados dos prontuários eletrônicos por meio do sistema informatizado do hospital. O conjunto de dados (sociodemográficos, atendimento inicial, condutas assistenciais à admissão, desfechos e o fluxo de identificação dos pacientes) derivados do prontuário eletrônico foi importado diretamente para o *Research Electronic Data Capture*.

### Análise dos resultados e estatísticas

O banco de dados foi exportado e submetido a uma verificação utilizando o *Microsoft Excel* ^®^, garantindo a identificação e correção de quaisquer inconsistências para assegurar a precisão dos dados antes da análise.

Devido à natureza exploratória do estudo e ao tamanho da amostra (n=58), optou-se pela realização de análises descritivas e bivariadas. As variáveis contínuas foram descritas por meio de medidas de tendência central e dispersão, utilizando-se a mediana, intervalo interquartil (IIQ), valores mínimos e máximos, devido à assimetria observada nas distribuições. A normalidade dos dados foi avaliada pelo teste de Shapiro-Wilk. Para as variáveis categóricas, realizaram-se frequências absolutas e relativas.

A comparação entre os grupos “identificada” e “não identificada” foi conduzida utilizando o teste qui-quadrado de Pearson, com aplicação do teste exato de Fisher em situações de baixa contagem esperada nas células. Para comparação de variáveis contínuas entre dois grupos independentes, empregou-se o teste de Mann-Whitney, considerando-se a ausência de distribuição normal. O nível de significância adotado em todas as análises foi de 5% (p<0,05).

## RESULTADOS

### Características sociodemográficas

Entre as 58 mulheres não identificadas admitidas no PS, obteve-se registro da idade de 41,3% delas, variando de 19 a 63 anos, com média de 32,21 (desvio padrão±12,50). Quanto à raça, informada por terceiros, a maioria (75,8%) foi classificada como parda. O local de residência foi registrado em 43,1% dos prontuários, sendo que mais da metade (60%) residia em Belo Horizonte. Dos 26 (44,8%) dados obtidos sobre o tipo de moradia, casa e apartamento foram majoritários (84,6%) (Tabela 1).

**Tabela 1:** Caracterização sociodemográfica de mulheres não identificadas admitidas no pronto-socorro, Belo Horizonte, Minas Gerais, Brasil, 2019-2022

Variável	n	%
**Idade (anos)**	**24**	**100**
18 a 29	13	54,17
30 a 39	4	16,67
40 a 49	4	16,67
50 a 59	2	8,33
60 ou mais	1	4,17
**Raça/cor**	**58**	**100**
Parda	44	75,86
Branca	7	12,07
Negra	7	12,07
**Cidade**	**25**	**100**
Belo Horizonte	15	60
Região Metropolitana	8	32
Minas Gerais, fora RMBH	2	8
**Tipo de moradia**	**26**	**100**
Casa/apartamento	22	84,62
Situação de rua	4	15,38

*RMBH - Região Metropolitana de Belo Horizonte.*

### Características da admissão no pronto-socorro

O mês de outubro se destacou pela maior frequência de admissões (17,2%), sendo os dias da semana com mais admissões os domingos (29,3%), seguidos pela terça-feira (20,6%) e por sábado (17,2%). Em relação ao turno de admissão, o período da madrugada se sobressaiu entre os demais, com 34,4%, e o período da noite representou 31%, seguido pelos períodos da tarde (24,1%) e manhã (10,3%).

Em relação à forma de chegada ao PS, a condução foi principalmente por equipes do Serviço de Atendimento Móvel de Urgência (48,2%), seguida por meios próprios (22,4%), órgãos de segurança pública (12%), ambulância particular (12%) e pela própria população (5,1%). No que diz respeito aos dados do SMCR, 57 (98,3%) das pacientes foram classificadas, sendo o nível de prioridade mais frequente laranja/muito urgente (63,2%), seguido por vermelho/emergência (21%). O fluxograma mais acessado foi trauma maior (40,3%), seguido por agressão (21%) (Tabela 2). Os discriminadores mais frequentes foram mecanismo de trauma significativo (36,8%), a junção de alteração do nível de consciência + alteração do nível de consciência não totalmente atribuível ao álcool + alteração do nível de consciência totalmente atribuível ao álcool (22,8%) e respiração inadequada (17,5%) (Tabela 2).

**Tabela 2:** Dados da classificação de risco segundo o Sistema Manchester de Classificação de Risco de mulheres não identificadas admitidas no pronto-socorro, Belo Horizonte, Minas Gerais, Brasil, 2019-2022

Variável	n	%
**Nível de prioridade - resultado do SMCR**	**57**	**100**
Vermelho/emergência	12	21
Laranja/muito urgente	36	63,2
Amarelo/urgente	6	10,5
Verde/pouco urgente	3	5,3
**Fluxograma do SMCR**	**57**	**100**
Trauma maior	23	40,3
Agressão	12	21
Quedas	4	7
Mal-estar em adulto	4	7
Queimaduras	2	3,5
Trauma cranioencefálico	2	3,5
Convulsões	2	3,5
Embriaguez aparente	2	3,5
Problemas em extremidades	2	3,5
Alteração do comportamento	1	1,8
Autoagressão	1	1,8
Feridas	1	1,8
Overdose e envenenamento	1	1,8
**Discriminador do SMCR**	**57**	**100**
Mecanismo de trauma significativo	21	36,8
Alteração do nível de consciência + alteração do nível de consciência não totalmente atribuível ao álcool + alteração do nível de consciência totalmente atribuível ao álcool	13	22,8
Respiração inadequada	10	17,5
Dor intensa + dor moderada + dor leve recente	6	10,5
Início súbito	2	3,5
Deformidade grosseira	1	1,8
História de overdose ou envenenamento	1	1,8
História de trauma	1	1,8
Novo pulso anormal	1	1,8
Obstrução de vias aéreas	1	1,8
**Motivo que levou a paciente ao pronto-socorro**	**58**	**100**
Agressão	17	29,3
Acidente de trânsito	12	20,7
Queda	9	15,5
Atropelamento	8	13,8
Intoxicação exógena	5	8,7
Alteração do nível de consciência	3	5,1
Queimadura	2	3,4
Crise convulsiva	2	3,4

SMCR - Sistema Manchester de Classificação de Risco;

* Agressão inclui agressão física, ferimento por arma de fogo, ferimento por arma branca;

** Acidente de trânsito inclui acidente automobilístico, acidente de motocicleta, acidente de bicicleta.

O motivo mais relevante que levou as mulheres ao PS foi agressão (29,4%), incluindo agressão física (12,1%), ferimento por arma de fogo (5,2%) e ferimento por arma branca (12,1%). Em seguida, estão os acidentes de trânsito (20,7%), compreendendo acidente automobilístico (12,1%), acidente de motocicleta (6,9%) e acidente de bicicleta (1,7%) (Tabela 2).

### Desfecho do atendimento a mulheres não identificadas no pronto-socorro

Dos 58 casos analisados, foi possível registrar o desfecho de 57 (98,3%) mulheres. Mais da metade delas (54,4%) não necessitou de internação, apresentando tempo de permanência no PS entre zero e três dias (mediana de zero; IIQ de um dia). A alta direta do serviço foi o desfecho mais frequente, ocorrendo em 48,2% dos atendimentos, e 6,9% das pacientes necessitaram de acompanhamento ambulatorial. Entre os demais desfechos, 18,9% foram encaminhadas ao bloco cirúrgico; 10,3% foram internadas; 6,9% evoluíram a óbito; 6,9% solicitaram alta a pedido; 5,1% foram transferidas; e 1,7% evadiram.

### Processo de identificação das pacientes

O período de permanência das mulheres como “não identificadas” variou de zero a 16 dias, com mediana de zero dias e amplitude interquartil de um.

Quanto ao processo de identificação, 22 (37,9%) pacientes foram identificadas até o momento do desfecho hospitalar. Dessas, 17 (77,3%) foram identificadas ainda no PS. Os dados do processo de identificação estão apresentados na Tabela 3.

**Tabela 3:** Processo de identificação das mulheres não identificadas admitidas no pronto-socorro, Belo Horizonte, Minas Gerais, Brasil, 2019-2022

Variável	n	%
**Identificação em algum momento durante a permanência no PS**	**58**	**100**
Não	41	70,7
Sim	17	29,3
**Identificação até o momento do desfecho hospitalar**	**58**	**100**
Sim	22	37,9
Não	36	62,1
**Responsável pela identificação da paciente**	**22**	**100**
Familiares	16	72,7
Autoidentificação	3	13,6
Amigos	2	9,1
Serviço policial	1	4,6
**Método utilizado para identificar a paciente**	**22**	**100**
Apresentação de documento de identificação	19	86,4
Autodeclaração (pandemia)	3	13,6
**Setor no qual a paciente foi identificada**	**22**	**100**
Pronto-socorro	17	72,7
Bloco cirúrgico	2	13,6
Unidade de internação	2	9,1
CTI	1	4,5
**Motivos para não identificação**	**36**	**100**
Sem registro do motivo	15	41,7
Autodeclaração sem documento de comprovação	13	36,1
Óbito	5	13,9
Recusa da identificação	2	5,6
Recusa do acionamento da família	1	2,7

*PS - Pronto-socorro; CTI - Centro de Terapia Intensiva.*

Ao comparar os atendimentos de mulheres identificadas e não identificadas durante a permanência no PS, observou-se associação significativa entre a identificação e o desfecho clínico. As distribuições dos desfechos diferiram de forma estatisticamente significativa entre os grupos (χ^2^=15,696; p=0,047), conforme apresentado na Tabela 4.

**Tabela 4:** Associação entre identificação da paciente e desfecho no pronto-socorro, Belo Horizonte, Minas Gerais, Brasil, 2019-2022

Desfecho no pronto-socorro	Não identificada n (%)	Identificada n (%)	Total n (%)
Alta direta	26 (63,5)	6 (35,3)	24 (41,4)
Bloco cirúrgico	8 (19,5)	3 (17,6)	11 (19,0)
Unidade de internação	1 (2,4)	5 (29,4)	6 (10,3)
Transferência hospitalar	1 (2,4)	2 (11,8)	3 (5,2)
Óbito	3 (7,3)	1 (5,9)	4 (6,9)
Evasão	1 (2,4)	1 (5,9)	2 (3,4)
Prontuário sem desfecho	1 (2,4)	0 (0,0)	1 (1,7)
**Total**	**41 (100)**	**17 (100)**	**58 (100)**

## DISCUSSÃO

Identificou-se que a faixa etária mais afetada foi, majoritariamente, a de jovens adultas, e a maioria foi registrada por terceiros como raça parda. Essa faixa etária se explica porque as mulheres vítimas de violência geralmente se encontram na faixa de idade reprodutiva e produtiva^(15)^. Além disso, a Organização Pan-Americana da Saúde afirma que a desigualdade é o principal fator de risco para a violência contra as mulheres, o que explica como mulheres mais jovens, especialmente em países de baixa ou média renda, enfrentam maiores dificuldades econômicas e sociais^(16)^. Fatores como baixa escolaridade, falta de acesso a recursos financeiros e dependência de parceiros externos propiciam maior vulnerabilidade a abuso^(9,10)^.

Quanto à raça, estudo transversal, no qual foram utilizados dados do VIVA Inquérito nos anos de 2011, 2014 e 2017, mostrou que os resultados encontrados refletem a situação da população feminina brasileira, já que, em todas as três edições analisadas, a raça parda foi a principal acometida nos casos de agressões que necessitaram de atendimentos de urgência e emergência^(17)^. De forma geral, o elemento raça/cor é um importante fator na vida das mulheres, reforçando um contexto de desigualdade racial e vulnerabilidade social que reflete, de forma negativa, na saúde e no acesso a serviços médicos para essas pacientes^(10)^.

A elevada proporção de dados faltantes nos registros de idade e local de residências pacientes não identificadas também merece destaque. Esse achado reflete a fragilidade dos processos de registro e comunicação entre equipes, interferindo na continuidade assistencial e na vigilância epidemiológica. A ausência de informações básicas impede o reconhecimento de padrões territoriais de vulnerabilidade, dificultando o planejamento de políticas públicas de prevenção e proteção social. Além disso, compromete práticas de enfermagem baseadas na integralidade e na segurança do paciente, pois limita o conhecimento contextual necessário para decisões clínicas e sociais assertivas.

A maioria das pacientes foi classificada como urgência (prioridade laranja), indicando gravidade clínica ao chegar ao serviço. Tal perfil é consistente com estudos que apontam pacientes não identificados como grupo de maior risco e gravidade nas emergências^(2)^. Os principais fluxogramas e discriminadores no acolhimento foram relacionados a trauma e alteração do nível de consciência, frequentemente associados a episódios de agressão e uso de álcool^(18)^. A agressão foi o motivo mais frequente de atendimento, seguida por acidentes de trânsito e quedas. No caso das agressões, a literatura aponta a predominância de casos envolvendo parceiros íntimos, frequentemente com o uso de força física e, em menor proporção, armas de fogo ou objetos perfurocortantes^(9,19)^.

Diante desse cenário de alta gravidade e prevalência de causas externas, o papel da enfermagem se torna essencial desde o primeiro contato com a paciente. Os resultados evidenciam a relevância da enfermagem no acolhimento e na classificação de risco de mulheres não identificadas, especialmente diante da alta proporção de casos classificados como prioridade laranja e vermelha. No SMCR, a ausência de informações de identificação impõe desafios adicionais, exigindo escuta qualificada e observação criteriosa para evitar subestimação do risco^(18-20)^. Nesse contexto, o enfermeiro atua como elo entre o cuidado clínico e a proteção social, garantindo atendimento seguro, encaminhamento oportuno e articulação com a rede de apoio às mulheres em situação de vulnerabilidade^(15,21)^.

Desde a admissão até o desfecho no PS, apenas parte das mulheres foi devidamente identificada, enquanto a maioria permaneceu anônima durante todo o atendimento. A análise dos desfechos clínicos revelou que a ausência de identificação repercute negativamente no fluxo assistencial, pois mulheres não identificadas apresentaram maior proporção de altas diretas e de óbitos, bem como menor probabilidade de internação. Esse padrão não indica resolutividade, mas evidencia limitações do cuidado prestado a pacientes cuja identidade não é confirmada, seja pela gravidade clínica que impede a comunicação, seja por falhas institucionais nos processos de registro, acolhimento e articulação intersetorial. O anonimato, portanto, amplia a invisibilidade institucional e compromete a continuidade do cuidado, dificultando o acionamento de redes de apoio e a tomada de decisões clínicas mais abrangentes.

Sob a ótica da ética do cuidado, a ausência de identidade reconhecida implica não apenas um risco administrativo, mas uma ruptura na continuidade da assistência e na proteção social. A literatura de enfermagem destaca que o cuidado ético requer reconhecimento e responsabilização pelo outro, superando barreiras institucionais que reforçam desigualdades e invisibilidades^(7-9)^. Pacientes que deixam o serviço sem qualquer forma de identificação permanecem fora do alcance das redes de vigilância, proteção e acompanhamento, o que dificulta o acionamento de políticas públicas de enfrentamento à violência e de suporte psicossocial^(19,21)^.

Nesse contexto, a enfermagem tem papel central por estar na linha de frente do acolhimento, da escuta qualificada e da articulação intersetorial. O enfermeiro pode intervir por meio da triagem ampliada com enfoque social, do registro sistemático de sinais de vulnerabilidade e da notificação de casos suspeitos de violência, conforme preconizado pela Norma Técnica de Prevenção e Tratamento dos Agravos Resultantes da Violência Sexual contra Mulheres e Adolescentes^(21)^ e reforçado por estudos sobre o papel da enfermagem no enfrentamento da violência e na segurança do paciente^(21-23)^_._

A articulação com o serviço social e os dispositivos de proteção à mulher, mesmo após a estabilização clínica, é fundamental para garantir vinculação pós-alta e inserção na rede de atenção psicossocial e de enfrentamento da violência. Assim, a associação entre tempo sem identificação e menor chance de internação não apenas evidencia desigualdades no fluxo assistencial, mas também convoca a enfermagem a repensar suas práticas sob uma perspectiva ética e de justiça social, orientada pelos princípios de integralidade, equidade e segurança do cuidado^(5,7,8)^.

### Limitações do estudo

Este estudo apresenta algumas limitações que devem ser consideradas. Por ter sido realizado em um único hospital de grande porte e perfil especializado, os resultados não podem ser generalizados para outras realidades assistenciais. A natureza retrospectiva da pesquisa, baseada em prontuários eletrônicos, também impôs restrições, pois parte das informações clínicas e sociais estava registrada em formato de texto livre, o que dificultou a padronização e aprofundamento dos dados. Além disso, a escrita livre está sujeita a variações interpretativas, influenciadas pela subjetividade dos profissionais que realizam os registros, podendo introduzir viés nos achados.

Além disso, observou-se a presença de campos incompletos ou ausentes em variáveis essenciais, como idade e local de residência, o que reflete fragilidades nos processos de registro e pode ter limitado a completude da análise. Essa característica é inerente a estudos documentais, podendo introduzir viés de informação. Por outro lado, tais lacunas evidenciam um aspecto relevante do próprio objeto de estudo: a precariedade dos registros sobre mulheres não identificadas que, por si só, reforça a discussão sobre vulnerabilidade e invisibilidade no contexto hospitalar.

### Contribuições para a área da enfermagem, saúde ou políticas públicas

O presente estudo contribui significativamente ao evidenciar uma temática ainda pouco abordada no campo da urgência e emergência: o atendimento a mulheres não identificadas. Ao descrever as características clínicas, sociais e de fluxo assistencial dessa população, a pesquisa amplia a compreensão sobre suas vulnerabilidades e lacunas de cuidado, especialmente nos processos de acolhimento e classificação de risco.

Os achados reforçam a centralidade do enfermeiro na triagem, no julgamento clínico e na tomada de decisão em contextos de urgência, nos quais a ausência de identificação demanda sensibilidade ética e discernimento técnico. A predominância das prioridades laranja e vermelha, associadas a atendimentos por agressão e trauma, indica que o enfermeiro é frequentemente o primeiro a reconhecer sinais de violência e vulnerabilidade, acionando protocolos de segurança, registro e encaminhamento à rede de proteção. Assim, o estudo oferece subsídios para o aprimoramento de protocolos assistenciais e fortalecimento de políticas públicas voltadas à segurança do paciente e à atenção integral à mulher, reafirmando o compromisso da enfermagem com um cuidado equitativo, ético e centrado nas necessidades humanas, mesmo diante da ausência de informações básicas como nome ou documento.

## CONCLUSÕES

As mulheres não identificadas atendidas em PS público apresentaram, majoritariamente, perfil de jovens adultas, de raça parda, com atendimentos mais frequentes durante a madrugada e aos finais de semana. Os casos de agressão e acidentes foram as principais causas de admissão, e a maioria das pacientes foi classificada como prioridade 2 (laranja/muito urgente) ou 1 (vermelha/emergência), indicando elevado risco clínico e necessidade de intervenção imediata. Observou-se que ausência de identificação repercute negativamente no fluxo assistencial, pois mulheres não identificadas apresentaram maior proporção de altas diretas e de óbitos e menor probabilidade de internação.

Os achados evidenciam que a ausência de identificação ultrapassa uma questão administrativa, configurando-se como marcador de vulnerabilidade social e assistencial que compromete a segurança e continuidade do cuidado. Nesse contexto, o enfermeiro exerce papel central no acolhimento e na classificação de risco, articulando julgamento clínico e sensibilidade para reconhecer sinais de violência, negligência e desproteção social. Tais resultados reforçam a necessidade de protocolos e fluxos institucionais que incorporem critérios clínicos e sociais ao processo de triagem, bem como estratégias de identificação seguras, ágeis e interdisciplinares. Em conjunto, esses elementos fortalecem a prática ética e humanizada da enfermagem, destacando que o cuidado de mulheres não identificadas exige abordagem integrada e orientada pela equidade.

## Data Availability

Os dados de pesquisa estão disponíveis em repositório: https://doi.org/10.48331/SCIELODATA.NH50AO.
